# Prediction of Mortality in Acute Thermal Burn Patients Using the Abbreviated Burn Severity Index Score: A Single-Center Experience

**DOI:** 10.7759/cureus.26161

**Published:** 2022-06-21

**Authors:** Amir Usmani, Dharmendra K Pipal, Harsh Bagla, Vijay Verma, Pawan Kumar, Seema Yadav, Garima Garima, Vibha Rani, Rajendra K Pipal

**Affiliations:** 1 General Surgery, Dr. Sampurnanand Medical College, Jodhpur, IND; 2 General, Colorectal and Minimal Access Surgery, All India Institute of Medical Sciences, Gorakhpur, Gorakhpur, IND; 3 Surgery, Dr. Sampurnanand Medical College, Jodhpur, IND; 4 Anaesthesia, Jaipur National University Medical College, Jaipur, IND; 5 Pathology, Government Medical College, Pali, Pali, IND; 6 Gynaecology and Obstetrics, All India Institute of Medical Sciences, Gorakhpur, Gorakhpur, IND; 7 Orthopaedics, Geetanjali Medical College, Udaipur, IND

**Keywords:** mortality, full-thickness burn, inhalational burns, total body burned surface area (tbsa), abbreviated burn severity index (absi) score

## Abstract

Background

Burn injuries are highly variable and dynamic. The outcome of patients is influenced by various factors and requires prompt therapeutic interventions, including fluid resuscitation, for a favorable result. Although having varying shortcomings, many scoring indexes are developed and validated in Western countries to predict mortality in a burn patient. The Abbreviated Burn Severity Index (ABSI) estimates survival expectancy in a burn patient via various negative prognostic factors. This study describes the pattern of burn injuries to validate the ABSI as an outcome predictor in burnt patients.

Methodology

From January to December 2018, 100 patients participated in this observational research conducted in the Department of Surgery at Mahatma Gandhi Hospital’s Burn Ward, a part of Dr. Sampurnanand Medical College, Jodhpur. Risk factors for death from a burn were patients’ age and gender, the depth of the burn, the inhalation burn, and the total burned body surface area (TBSA). The area under the receiver operating curve (AUROC) was used to determine how well it could predict burn deaths.

Results

This study included 100 patients (69 men and 31 women, with a ratio of 2.22:1). In total, 73 patients survived, and 27 died (a mortality rate of 27%). The fatality rate increased with increased burn surface area, reaching 100% in patients with >80% burns (p < 0.0001). Additionally, those with an ABSI of >11 expressed 100% mortality rate (p < 0.0001).

Conclusions

In this study, older age, high burned surface area, concomitant inhalational burns, full-thickness burns, and a higher ABSI were found to be significant predictors of mortality.

## Introduction

With an annual fatality rate of 2.1% per 100,000 cases in Western and other developed countries, burn injuries are categorized as a major cause of mortality and lifelong disability globally [1,2]. The socioeconomic conditions of burn patients and the patterns of burn injuries differ from place to place. Higher mortality rates can be explained by the higher rate of therapy restrictions for patients with self-inflicted burns [3]. Demographics, such as age and sex, the burned surface area, concurrent inhalation injury, and comorbid conditions, such as diabetes, co-existing trauma, and pneumonia, are risk factors for mortality [4]. Because of enhanced care in well-equipped critical care units, including skin banks and effective silver-impregnated bandages with early wound care, survival rates of burn injuries are improving in developed countries but not yet established in developing countries. Even though regular assessments are an important element of burn care, the treating surgeon is nonetheless interested in predicting the outcomes of acute burn patients. This evaluation is critical because it aids in triage, leads management decision-making, and allocates resources, which is especially crucial in resource-constrained situations in a developing country like India.

## Materials and methods

This prospective, observational study was conducted in the Department of Surgery at Mahatma Gandhi Hospital’s Burn Ward, a part of Dr. Sampurnanand Medical College, Jodhpur, India. In addition to monitoring vital signs and oxygen saturation, fluid resuscitation was calculated using the Parkland equation [5]. Major burns were defined as those that covered 15% or more of the total body surface. Using Table 1, the Abbreviated Burn Severity Index (ABSI) was scored into six groups, namely, 2-3, 4-5, 6-7, 8-9, 10-11, and ≥12 [6]. Expected survival related to ABSI score (Table 2) was used to correlate actual survival to ABSI score. The outcomes included mortality and discharge from the hospital.

**Table 1:** Calculation of the Abbreviated Burn Severity Index score. BSA: body surface area

Parameter	Finding	Points
Sex	Female	1
	Male	0
Age (years)	0–20	1
	21–40	2
	41–60	3
	61–80	4
	81–100	5
Inhalation injury	Yes	1
	No	0
Presence of full-thickness burn	Yes	1
	No	0
BSA burn (%)	1–10	1
	11–20	2
	21–30	3
	31–40	4
	41–50	5
	51–60	6
	61–70	7
	71–80	8
	81–90	9
	91–100	10

**Table 2:** The ABSI score and prediction. ABSI: Abbreviated Burn Severity Index

ABSI	Threat to life	Probability of survival (%)
2-3	Very low	≥99%
4-5	Moderate	98%
6-7	Moderately severe	80–90%
8-9	Serious	50–70%
10-11	Severe	20–40%
≥12	Maximum	≤10%

Inclusion and exclusion criteria

All thermal burn patients (second and third-degree burns) who were admitted from January to December 2018 to the Burn Unit under the Department of General Surgery. Those who had chemical, electrical, or radiation burns, as well as comorbidities such as diabetes, malignancy, or immunocompromised state, other trauma, pregnant women, patients who had a 24-hour gap between exposure and admission, and patients who did not want treatment were excluded.

After applying the abovementioned inclusion and exclusion criteria, 100 patients were found suitable to include in this study.

Data collection

**Figure 1:**
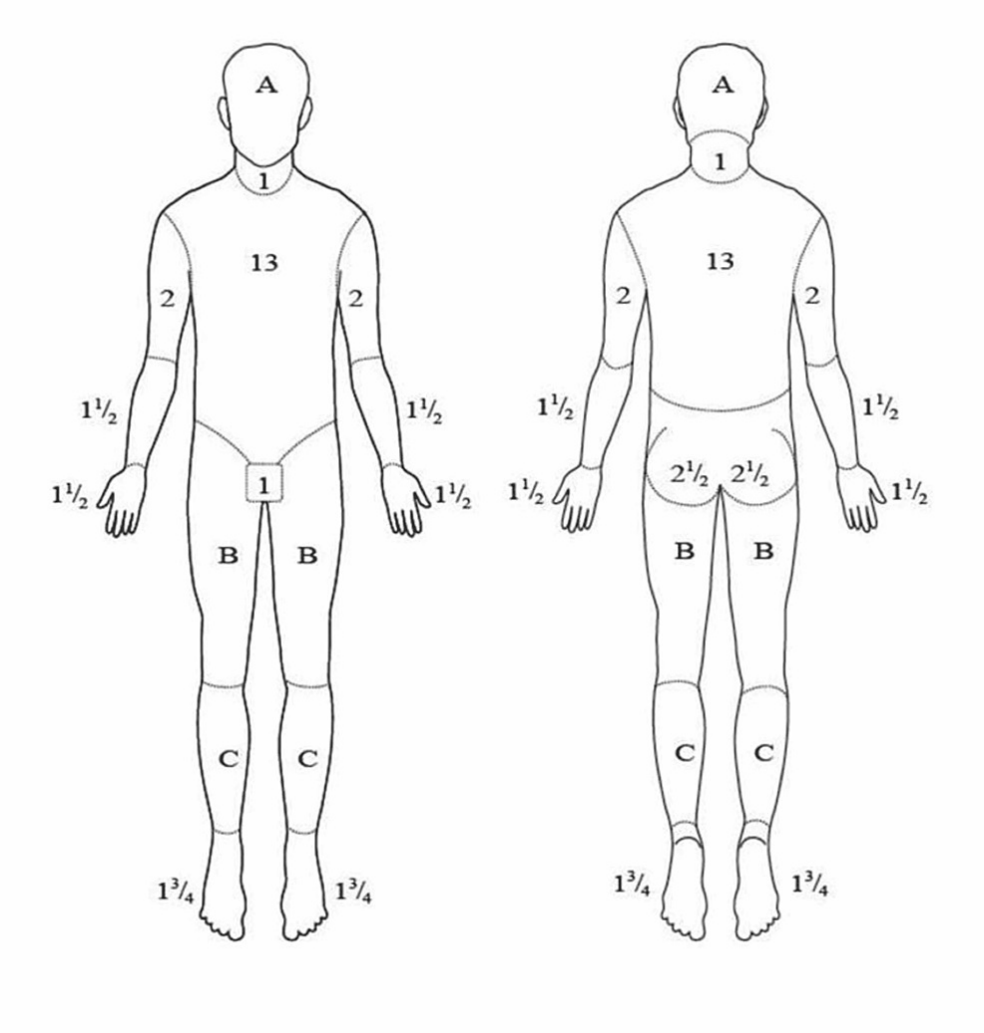
Calculation of total burn surface area using Lund and Browder’s chart.

After taking down patients’ information, a physical examination for full-thickness burns and concomitant inhalation burns was performed. Two of each physical sign and symptom, with or without a history of close space fire, were required to suggest inhalational injury. Symptoms included dyspnea, cough, wheezing, retractions, hoarseness, irritability, and temporal headache. Facial burns, blistering or edema of the oropharynx, singeing of hair, stridor, upper airway mucosal lesions, carbonaceous sputum, rales, rhonchi, decreased breath sounds, coma (nearly always from CO poisoning). The Lund and Browder chart (Figure 1) was used to calculate burnt body surface area. and in children, body surface area (BSA) burn (%) was computed using the criteria presented in Table 3 [7,8]. The degree of burn, whether full-thickness or partial-thickness, was categorized according to Table 4 [9]. The above data were used to compute the ABSI score.

**Table 3:** Calculation of the total burn surface area in children.

Area	Age (years)					Adult
	0–4	1–4	5–9	10–14	15	
A = half of head	9½	8½	6½	5½	4½	3½
B = half of one thigh	2¾	3¼	4	4½	4½	4¾
C = half of one lower leg	2½	2½	2¾	3	3¼	3½

**Table 4:** Categories of burn depth.

Burn degree	Surface appearance	Color	Pain level
First (superficial)	Dry, no blisters, no or minimal edema	Erythematous	Painful
Second (partial-thickness)	Moist blebs, blisters	Mottled white to pink, cherry red	Very painful
Third (full-thickness)	Dry with leathery eschar until debridement, charred vessels visible under eschar	Mixed white, waxy, pearly; dark, khaki, mahogany; charred	Little or no pain; hair pulls out easily

The ABSI score was calculated as follows: ABSI score = Age + Sex + Presence of full-thickness burn + TBSA + Presence of inhalational injury.

The data collected during the study were compiled and statistically analyzed using the SPSS version 22.0 software package (IBM Corp., Armonk, NY, USA). Qualitative data were expressed as numbers and percentages for categorical variables, while quantitative data were expressed as mean and standard deviations for continuous variables. The difference in proportion was analyzed using the chi-square test. The difference in mean among the groups was analyzed using the Student’s t-test and coefficient correlation. The study was approved by the Institutional Review Board Committee (approval number: F.1/Acad/MC/JU/18/6878). P-values of <0.05 were considered statistically significant.

## Results

Of the total 100 patients, 73 survived and 27 died. As the age advanced, the mortality rate increased (p < 0.005). There were 69 male and 31 female patients in this study at a ratio of 2.22:1 (Table 5). Male patients had a mortality rate of 5.55%, whereas female patients had a mortality rate of 20% (p = 0.05).

**Table 5:** Correlation between sex and mortality among study patients.

Sex	Number of patients	Percentage	Discharge	Expired	Percentage mortality	P-value
Male	69	60	55	14	20.29	0.03
Female	31	40	18	13	41.94	
Total	100	100	73	27	100	

The majority of the females were between the ages of 14 and 36 years, with a mean age of 31.28 years. Overall, 13 of the 31 women died (41.94%, p = 0.05). The majority of the males were between the ages of 20 and 40, with a mean age of 26.60 (Table 6). The total number of elderly patients was 5 (5%), and they all died, resulting in a 100% mortality rate.

**Table 6:** Demographic profile of patients and correlation between age and mortality.

Age (years)	Number of patients	Discharge	Expired	P-value
≤20	34 (34%)	31 (91.17%)	3 (8.82%)	<0.001
21–40	49 (49%)	38 (77.55%)	11 (22.45%)	
41–60	12 (12%)	4 (33.33%)	8 (66.67%)	
61–80	3 (3%)	0 (0%)	3 (100%)	
≥81	2 (2%)	0 (0%)	2 (100%)	
Total	100 (100%)	73 (73%)	27 (27%)	

In our study, patients were admitted with burned surface areas of 6% to 96% (Table 7). The mean body burned surface area was 37.7%. Burned surface areas of >80% (100%) accounted for the highest mortality, while burned surface areas of 11-20% were associated with the lowest mortality (0%).

**Table 7:** Correlation between the total burn surface area and mortality. TBSA: total body surface area

TBSA (%) Mean ± 37.7	Number of patients	Discharge	Expired (% mortality)	P-value
≤10	8 (8%)	7 (87.5%)	1 (12.50%)	<0.001
11–20	13 (13%)	13 (100%)	0 (0.00%)	
21–30	19 (19%)	18 (94.73%)	1 (5.26%)	
31–40	26 (26%)	22 (84.62%)	4 (15.38%)	
41–50	9 (9%)	4 (44.44%)	5 (55.56%)	
51–60	11 (11%)	6 (54.55%)	5 (45.45%)	
61–70	6 (6%)	2 (33,33%)	4 (66.67%)	
71–80	4 (4%)	1 (25%)	3 (75%)	
81–90	3 (3%)	0 (0.00%)	3 (100%)	
91–100	1 (1%)	0 (0.00%)	1 (100%)	
Total	100 (100%)	73 (73%)	27 (27%)	

Total inhalational burns were 37, and the mortality associated with them was 16 (43.24%) (p < 0.05). Patients with full-thickness burns were 38 and the mortality was 18 (47.36%) (p < 0.05) (Table 8). Mortality in patients with partial-thickness burns was nine (14.51%).

**Table 8:** Correlation of Inhalational burn and degree of burn with mortality.

Variable		Number of patients (%)	Discharged	Expired	P-value
Inhalational injury	With inhalation	37 (37%)	21 (21%)	16 (43.24%)	0.008
	Without inhalation	63 (63%)	52 (52%)	11 (17.46%)	
Total		100 (100%)	73 (73%)	27 (27%)	
Degree of burn	Full-thickness	38 (38%)	20 (52.63%)	18 (47.36%)	0.002
	Partial-thickness	62 (62%)	53 (85.48%)	9 (14.52%)	
Total		100 (100%)	73 (73%)	27 (27%)	

In our study, minimum mortality was noted among patients with ABSI scores of 2 to 3, and the maximum mortality was noted at a score of >11 (Table 9). The cutoff value of the ABSI score for mortality was 10.59. The score with minimum mortality was 2. The mean ABSI score was 7.29. Mortality increased with the score, and the survival was 100% with a score of 2-3. On the other hand, survival was zero at a score of >11 (p = 0.001), which was highly significant.

**Table 9:** Correlation between the ABSI score and mortality. ABSI: Abbreviated Burn Severity Index

ABSI score (Mean = 7.29)	Number of patients (%)	Discharged	Expired (% mortality)	Expected survival (%)	P-value
2-3	3 (3%)	3 (100%)	0 (0.00%)	>99%	<0.0001
4-5	30 (30%)	29 ((96.67%)	1 (3.33%)	98%	
6-7	32 (32%)	28 (87.5%)	4 (12.50%)	80–90%	
8-9	13 (13%)	11 (84.62%)	2 (15.38%)	50–70%	
10-11	12 (12%)	2 (16.67%)	10 (83.33%)	20–40%	
≥12	10 (10%)	0 (0%)	10 (100%)	<10%	
Total	100 (100%)	73 (73%)	27 (27%)		

The ABSI score had at least one tie between the positive actual state group and the negative actual state group.

**Figure 2:**
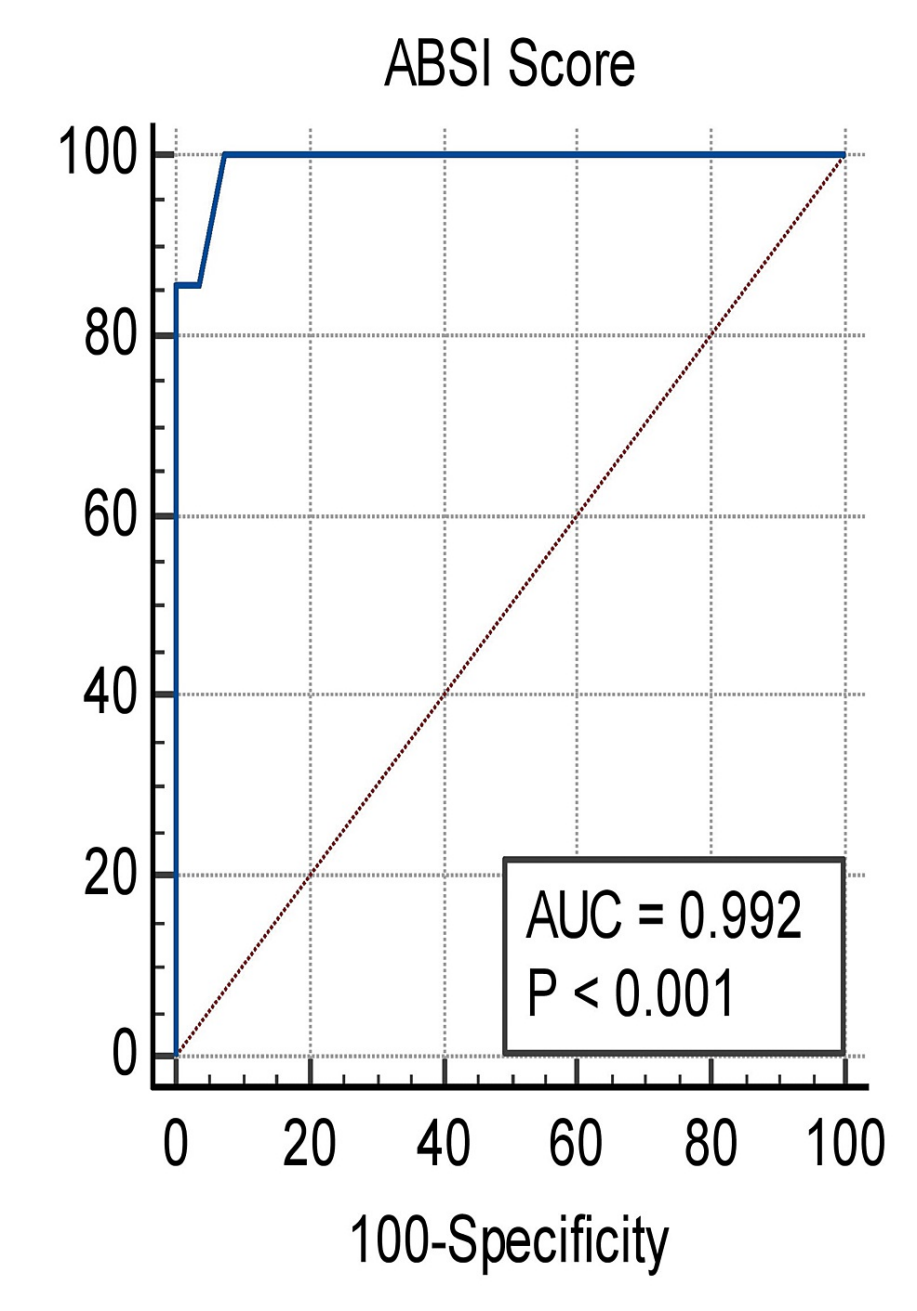
Receiver operating curve of ABSI to show the relationship between sensitivity and specificity. AUC: area under the curve; ABSI: Abbreviated Burn Severity Index

Classifiers with curves closer to the top-left corner indicate better performance; hence, ABSI can be considered a good predictor. Moreover, greater AUC concurs with predictive accuracy. At the criterion value of >8, sensitivity was 1 and specificity was 0.92. (Table 10, Figure 2).

**Table 10:** Criterion values and coordinates of the ROC curve. CI: confidence interval; LR: likelihood ratio; ROC: receiver operating curve

Criterion	Sensitivity	95% CI	Specificity	95% CI	+LR	-LR
≥4	100.00	59.0–100.0	0.00	0.0–12.8	1.00	
>8	100.00	59.0–100.0	92.59	75.7–99.1	13.50	0.00
>9	85.71	42.1–99.6	96.30	81.0–99.9	23.14	0.15
>10	85.71	42.1–99.6	100.0	87.2–100.0		0.14
>13	0.00	0.0–41.0	100.0	87.2–100.0		1.00

## Discussion

Elderly patients (>60 years, p < 0.05) had the highest mortality rate, whereas those under 20 years of age had the lowest, accounting for 8.82% of the total in this study. However, males had more burn injuries in all demographic groups, yet females had a higher mortality rate, except for those over the age of 59. According to Rani and Schwacha in 2012 [10], age-related altered immunity in elderly patients may predispose them to more infections and poorer healing. According to McGwin et al., the female mortality rate was more than twice as high as that of males (odds ratio (OR) = 2.3) despite similar causes and timing of mortality among burn patients up to the age of 60 [11]. A study of 4,927 patients by O'Keefe et al. [12] reported that women aged between 30 and 59 years had a two-fold higher risk of mortality than men of a similar age group. Kerby et al. [13] noted that female burn patients had a 30% higher risk of dying than male burn patients. The patients with the maximum number (49%) were in the age range of 21-40 (Table 6) in this study, possibly because this age group is more susceptible to occupational hazards, domestic violence, suicide, and homicides.

For a wound with equivalent severity, men were referred to a higher quality of healthcare more frequently than women. Despite possessing comparable ABSI scores, the results suggest that men and women have different dispositions. Female children were more likely than male children to die because of neglect, gender biases, domestic abuse, and poor nutritional status. This explains why girls in another age group have a greater mortality rate [14].

Because a disproportionate proportion of fire deaths occurs in houses, children aged 0-5 years are at higher risk. Size and body proportions are the most prominent disparities between adults and children. Some theories for the higher burn fatality rate in children include a lower physiologic reserve, thinner skin, complexities in obtaining cannulation, a narrower wiggle room for fluid management, and a stronger refrainment from traumatic debridement of the burn wound and other procedures. Because hormonal, as well as physiologic, differences are comparable for both men and women, gender appears to have little impact on outcomes, including mortality in the elderly. They are both susceptible to risk factors and debilitating conditions, such as malnutrition, lean body mass, old skin, or wound healing, which can make it more likely that the elderly will die than younger people [15].

The degree of full-thickness burn combined with flame and scald appeared to have no statistically significant difference. The duration and temperature of hot liquid or flame in contact with the body surface are related to the degree of burn-in scald or flame burn. Inhalational burns occur when noxious vapors are inhaled, which is common in flame burns.

Male mortality was 14 (20.29%) with a p-value of <0.05. The majority of males were between the ages of 21 and 40, with a mean age of 26.60 years. Among females, mortality was 13 out of 31 (41.94%). Bhansali et al. [16] concluded that female patients have a higher mortality rate compared to males. As shown in Table 6, the maximum mortality (100%) associated with burn surface area was greater than 80%, while the minimum mortality (p < 0.05) was observed in 20% of the TBSA group. In 2007, Jeschke et al. [13] concluded that the percentage of predicted resting energy expenditure was the highest in the >80% TBSA group, followed by the 60-79% TBSA burn group (p < 0.05).

An Indian study observed 100% mortality in those who had more than 60% of total burn surface [17]. Similarly, our study had the same observation, i.e., 100% mortality in those with a higher burn surface (Table 7). Regarding the extent of the burn and age, Bull et al. [18] considered these factors the most crucial variables in determining if a patient lived or died. Our current research corroborates this observation, with worse life expectancy among the elderly and more total surface areas burned. This effect was more pronounced at the extremes of age due to poor physiological reserve. An increase in TBSA led to increased hypermetabolism and catabolism. Small burns lead to little mortality, and as the size of the burn advances, mortality also increases to a substantial degree before plateauing at its maximum of 100%.

In our study, according to Table 8, patients who presented with inhalational burns sustained higher mortality than those who did not (43.24% vs. 17.46%), with a p-value of <0.05. In our study, inhalational burns were most common between 20 and 40 years of age. The inhalation of the products of combustion can lead to devastating pulmonary injury, which significantly increases burn mortality for a given percentage of skin burns. In these patients, carbon monoxide inhalation is potentially devastating.

After a subglottic inhalation injury, the predominant pathophysiologic alteration is an augmented bronchial blood supply. Bronchial edema develops due to mucosal hyperemia, excessive mucous secretion, and plasma transudation and leads to poor ventilation by blocking the airway, which is further aggravated by inspissated mucous admixed with fibrin clots and epithelial debris. This warrants prompt bronchoscopy and multidisciplinary management [19]. Considering the lack of globally accepted standard treatments, there is enough evidence to support the use of bronchodilators, bronchoscopic removal of the mucous cast by suction, use of aerosolized heparin, and nebulization with N-acetylcysteine for dissolution of fibrin and mucus casts [19].

At the time of presentation, the depth of the burn was assessed as per Table 4. We observed that full-thickness burns were positively associated with higher fatalities than partial-thickness burns (Table 9), with a significant p-value. Chen et al. [20] reported that unless a patient does not have an inhalation injury, the estimated fatality rate with a second-degree burn injury was 4.82%, and with a third-degree burn injury was 20.50%. With the increase in depth of burns, there is increased inflammation, hypermetabolism, and the need for surgical interventions, which carry surgical as well as anesthetic risks. Full-thickness burns must be referred to a highly dedicated burn center as early as possible because they are associated with the loss of regenerative elements in the wound, thus, taking many weeks to heal with a high propensity to dense contracture [21,22].

Three weeks is unlikely to be enough time for deep burns to heal. If healing is protracted from three to six weeks, the probability of ugly hypertrophic and thick scarring increases from 33% to 78% [22]. Consequently, within the first 5-10 days, such wounds should also be dissected and grafted. At ABSI scores of 8-9 and 10-11, survivorship dropped considerably from 84.62% in the former to 16.67% in the latter (Table 9). To improve their survival, patients in ABSI categories 10-11 require more rigorous intervention. These results imply that ABSI has a high level of precision in anticipating mortality.

Thus, according to data analysis, the sensitivity was 0.96 and the specificity was 1 (Figure 2, Table 10). There is at least one tie in the ABSI score between the positive and negative real state groups. These findings corroborated those of Gutierrez et al. [23], who conducted a retrospective study on burns. A retrospective study of burns, conducted by Nthumba et al. [24], observed that overall survival decreased considerably between ABSI scores of 6-7 and 8-9 from 70% in the former to 20% in the latter.

The current study is limited, and a larger study should be conducted to determine the viability of ABSI score in acute thermal burn patients.

## Conclusions

The ability to predict the outcomes of extensive burns is critical for informing clinical and economical decisions that benefit patients’ families and medical providers. The ABSI score system is extremely straightforward to use and calculate, making it a reliable and simple technique for predicting burn injury fatality. With the results of our research, we conclude that the ABSI is a candid and pragmatic scoring system that accurately predicts mortality in burn patients.
